# Real‐world data prognostic model of overall survival in patients with advanced NSCLC receiving anti‐PD‐1/PD‐L1 immune checkpoint inhibitors as second‐line monotherapy

**DOI:** 10.1002/cnr2.1578

**Published:** 2022-01-24

**Authors:** Cristina Julian, Robson J. M. Machado, Sandhya Girish, Pascal Chanu, Dominik Heinzmann, Chris Harbron, Anda Gershon, Shannon M. Pfeiffer, Wei Zou, Valerie Quarmby, Qing Zhang, Yachi Chen

**Affiliations:** ^1^ Genentech, Inc South San Francisco California USA; ^2^ Roche Products Welwyn Garden City UK; ^3^ Genentech/Roche Lyon France; ^4^ F Hoffmann‐La Roche Ltd Basel Switzerland; ^5^ Present address: Drax Group plc Shelby UK; ^6^ Present address: Gilead South San Francisco California USA; ^7^ Present address: The Parker Institute for Cancer, Immunotherapy San Francisco California USA

**Keywords:** advanced NSCLC, immune checkpoint inhibitors, prognostic model, real‐world data

## Abstract

**Background and aim:**

The objective of this retrospective, observational, noninterventional cohort study was to investigate prognostic factors of overall survival (OS) in patients with advanced non‐small cell lung cancer (aNSCLC) and to develop a novel prognostic model.

**Methods:**

A total of 4049 patients with aNSCLC diagnosed between January 2011 and February 2020 who received atezolizumab, nivolumab, or pembrolizumab as second‐line monotherapy were selected from a real‐world deidentified database to build the cohort. Patients could not have received first‐line treatment with clinical study drug(s) nor immune checkpoint inhibitors including anti‐programmed cell death 1 (PD‐1)/programmed death‐ligand 1 (PD‐L1), and anti‐cytotoxic T‐lymphocyte‐associated protein 4 therapies.

**Results:**

Patients had a median age of 69 years; 45% were female, 75% White, 70% had stage IV at initial diagnosis, and 70% had nonsquamous histology. A Cox proportional hazards model with lasso regularization was used to build a prognostic model for OS using 18 baseline demographic and clinical factors based on the real‐world data cohort. The risk‐increasing prognostic factors were abnormally low albumin and chloride levels, Eastern Cooperative Oncology Group performance status score ≥ 2, and abnormally high levels of alkaline phosphatase and white blood cells. The risk‐decreasing prognostic factors were PD‐L1 positivity, longer time from advanced diagnosis to start of first‐line therapy, and higher systolic blood pressure. The performance of the model was validated using data from the OAK trial, and the c‐index for the OAK trial validation cohort was 0.65 and 0.67 for the real‐world data cohort.

**Conclusions:**

Based on baseline demographic and clinical factors from a real‐world setting, this prognostic model was developed to discriminate the risk of death in patients with aNSCLC treated with checkpoint inhibitors as second‐line monotherapy, and it performed well in the real‐world data and clinical trial cohorts.

## 1 INTRODUCTION

Lung cancer is the most prevalent type of cancer worldwide. It is estimated that lung cancer contributes to 24% of cancer‐related deaths in men and 23% of cancer‐related deaths in women in the US.^1^ Survival outcomes of patients with lung cancer have improved due to the development of new treatments such as immunotherapy. This improvement is especially pronounced in patients with non‐small cell lung cancer (NSCLC).^2^ Use of immune checkpoint inhibitors (CPIs) as second‐line (2L) treatment for patients with advanced NSCLC (aNSCLC) has improved survival and antitumor response compared with chemotherapy. Specifically, monoclonal antibodies against programmed cell death 1 (PD‐1; nivolumab and pembrolizumab) and programmed death‐ligand 1 (PD‐L1; atezolizumab) as 2L monotherapy have shown a benefit in overall survival (OS) compared with docetaxel.^3^, ^4^, ^5^ However, despite this increase in survival benefit, 40% to 60% of patients do not respond to these therapies.^3^, ^5^, ^6^, ^7^

Research on prognostic factors in patients with NSCLC receiving CPIs in 2L treatment has mainly focused on one or a few risk factors, such as PD‐L1 expression that has been shown to be prognostic for PD‐L1 agents in NSCLC.^8^ Although PD‐L1 expression can help physicians decide on appropriate treatment for a patient, a prognostic model accounting for multiple factors simultaneously could create clinical risk groups for stratifying patients by disease severity. One multivariable prognostic risk model for OS, the Real wOrld PROgnostic (ROPRO) score, was derived across 17 cancer cohorts from 27 demographic, clinical, and routine laboratory parameters and validated using two independent phase I and III trials.^9^ In the NSCLC cohort, patients with higher ROPRO scores (upper 10%) had an almost eightfold increased risk of death compared with patients with lower scores (lower 10%).^9^ While this model can be applied to different cancer indications, a model with a smaller number of variables tailored to a specific indication, such as NSCLC, may facilitate its application and also minimize overall missing data by using less variables. The objective of this study was to build a prognostic model using clinical and laboratory factors that are prognostic of OS in patients with aNSCLC who received an anti‐PD‐1/PD‐L1 CPI as 2L monotherapy in a real‐world setting. An independent clinical trial cohort was used to validate the model.

## 2 METHODS

### 2.1 Training cohort

This retrospective, observational, noninterventional cohort study used the nationwide Flatiron Health electronic health record–derived deidentified database. During the study period, the deidentified data originated from ≈280 US cancer clinics (≈800 sites of care).

The Flatiron Health database is longitudinal and consists of deidentified, patient‐level, structured and unstructured data, curated via technology‐enabled abstraction.^10^, ^11^ Patients with aNSCLC diagnosed between January 1, 2011, and February 1, 2020, who received anti‐PD‐1/PD‐L1 CPIs (atezolizumab, nivolumab, or pembrolizumab) as 2L monotherapy were included. Patients were aged ≥18 years at the time of advanced diagnosis, not previously diagnosed with other types of cancer, had started any treatment within 90 days after advanced diagnosis, and could not have received first‐line (1L) treatment with clinical study drug(s) nor CPIs including anti‐PD1/PD‐L1 or anti‐cytotoxic T‐lymphocyte‐associated protein 4. Patients were followed until death, last observed patient activity, or the database cutoff date (February 1, 2020).

### 2.2 Prognostic factors

A literature review on relevant prognostic factors was conducted using PubMed over the past 10 years to identify reviews and trials that evaluated prognostic factors of OS or progression‐free survival in patients with lung cancer receiving CPIs.^7^, ^8^, ^12^, ^13^, ^14^, ^15^ Primary variables of interest in the real‐world database included demographic, clinical, and laboratory characteristics that were measured at baseline.

Treatment start was defined as the first date of drug administration in 2L therapy. Sex, age at advanced diagnosis, and age at start of 2L therapy were included in the model. Race was categorized as Asian, Black or African American, Hispanic or Latino, White, or other. Smoking status was defined by previous/current or never smoker. Eastern Cooperative Oncology Group performance status (ECOG PS) scores were used to determine the patient's performance status and were categorized as 0/1 and ≥ 2. NSCLC stage at initial diagnosis was included in the model and categorized as stage <IV or IV. Other disease‐related variables included histology, body mass index, systolic blood pressure, diastolic blood pressure, tumor PD‐L1 expression (positive, ≥1%; negative, <1%), and mutation status for *KRAS* and *EGFR*.

Laboratory values were determined using standard clinical chemistry methods and then classified as abnormally low, within the normal range, or abnormally high (except for albumin, which was classified as abnormal [low] and normal). The following parameters were assessed: serum albumin, circulating monocyte, lymphocyte, thrombocyte, neutrophil, and platelet counts, white blood cell counts, absolute eosinophil counts, bilirubin, lactate dehydrogenase, alkaline phosphatase (ALP), aspartate aminotransferase, alanine transaminase, chloride, calcium, creatinine, total serum protein, urea, and hemoglobin. The interactions of neutrophils and lymphocytes, lymphocytes and monocytes, platelets and lymphocytes, neutrophils and white blood cells, and aspartate aminotransferase and alanine transaminase were also investigated. Reference ranges vary across laboratories and were based on instrument sensitivity as well as units of measure for the particular test at the performing laboratory.

### 2.3 Validation cohort

The developed real‐world data (RWD) prognostic model was applied retrospectively to data from the OAK trial (blinded for peer review), a phase III, open‐label, randomized controlled trial that assessed the efficacy and safety of atezolizumab monotherapy compared with docetaxel as 2L treatment in patients with aNSCLC.^7^ The cohort from the OAK trial was selected based on the following criteria: squamous or nonsquamous NSCLC, age ≥ 18 years, measurable disease per Response Evaluation Criteria in Solid Tumors version 1.1, ECOG PS 0/1, previous chemotherapy, and no prior treatment with PD‐1/PD‐L1 inhibitors.

### 2.4 Statistical analysis

A Cox proportional hazards model with lasso regularization was used to build the prognostic model based on the aforementioned demographic, clinical, and laboratory factors to select variables that contribute most to OS prediction.^16^ The model was estimated using the GLMNET package in R with *α* = 0.9 and λ tuned using a grid search. The prognostic index (PI) was fitted to predict the risk of death for a patient. The PI was calculated based on the following Cox model:

ℎ_i_(t) = ℎ_0_(t) exp(𝛽^T^ *x*_*i*_),where *h*_i_(t) is the hazard function for a patient, ℎ_0_(t) is an unspecified baseline hazard function, *x*_i_ is a p × 1 vector of covariates for that patient and 𝛽 is a p × 1 vector of coefficients. The number 𝛽^*T*^ *x*_*i*_ was defined as the PI. Larger values of PI implied shorter survival times, while smaller values of PI implied longer survival times. Survival data were represented by quartiles of PI values. A 10‐fold cross‐validation with a split of 80% for the training set and 20% for the testing set was performed.

The validity of the model developed from the real‐world database was tested in the OAK data by assessing the model's discrimination. Kaplan–Meier OS curves, stratified into quartiles by the PI, were generated for both the RWD and trial cohorts. A concordance index (c‐index), defined as the proportion of concordant pairs divided by the total number of possible evaluation pairs, was then calculated for both the real‐world database and OAK.

### 2.5 Missing data

Missing values were imputed using the chained equation method and predictive mean matching as previously described in the literature.^17^ The imputation model included all variables used in the analysis model, including the outcome variables. In this case, the cumulative hazards and censor indicator were used for each patient.^18^ Given that the real‐world database and OAK data sets have different missing data mechanisms, separate imputation models for each data set were developed, with exactly the same model specification.

## 3 RESULTS

### 3.1 Patient characteristics

**FIGURE 1:**
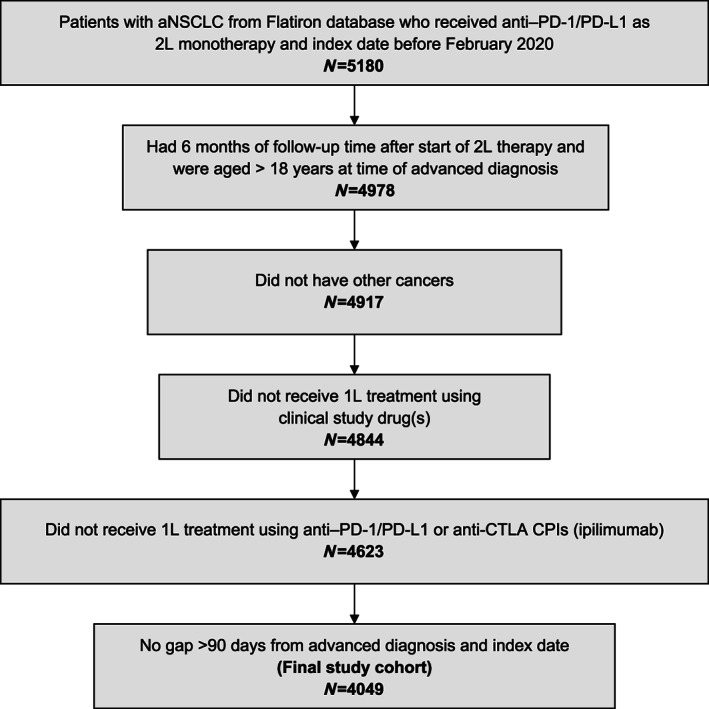
Patient flowchart. 1L, first line; 2L, second line; aNSCLC, advanced non‐small cell lung carcinoma; CPI, checkpoint inhibitor; CTLA, cytotoxic T‐lymphocyte‐associated protein; PD‐1, programmed cell death‐1; PD‐L1, programmed death‐ligand 1

A total of 4049 patients from the real‐world database and 792 patients from the OAK trial were included. Initially, 5180 patients with aNSCLC were selected from the RWD who had received anti‐PD‐1/PD‐L1 CPIs as 2L treatment and diagnosed between January 1, 2011, and February 1, 2020 (Figure 1).

Patients selected from the RWD had a median age of 69 years, 70% had stage IV NSCLC at diagnosis, and 70% had nonsquamous histology (Table 1). In the validation cohort from the OAK trial, median age was 63 years, ECOG PS was 0/1, for all patients per the trial inclusion parameters, and 74% had nonsquamous histology. The majority of patients in the RWD and OAK cohorts had laboratory values in the normal range, except the majority of patients in both cohorts having low hemoglobin levels and most patients in OAK had high calcium and urea levels (Table 2).

**TABLE 1:** Demographic and clinical characteristics of the patients included in the prognostic model

Characteristic	Real‐world database (*n* = 4049)	OAK trial (*n* = 792)
Age at index, median (IQR), years	69.0 (61.0, 76.0)	63.0 (57.0, 70.0)
Age at advanced diagnosis, median (IQR), years	68.0 (60.0, 75.0)	63.0 (56.0, 69.0)
Time from advanced diagnosis to index date, median (IQR), months	7.88 (4.93, 14.36)	13.52 (8.28, 24.44)
Heart rate, median (IQR), bpm	87.0 (76.0, 100.0)	82.0 (72.0, 93.0)
Diastolic blood pressure, median (IQR), mmHg	72.0 (66.0, 80.0)	75.0 (68.0, 80.0)
Systolic blood pressure, median (IQR), mmHg	123.0 (110.0, 137.0)	125.0 (114.0, 135.0)
Smoking status, *n* (%)		
Previous/current	3685 (91.0)	651 (82.2)
Never	364 (9.0)	141 (17.8)
Stages, *n* (%)		
Stage <IV	1211 (29.9)	320 (40.4)
Stage IV	2837 (70.1)	472 (59.6)
Sex, *n* (%)		
Female	1834 (45.3)	309 (39.0)
Male	2215 (54.7)	483 (61.0)
Histology, *n* (%)		
Nonsquamous	2826 (69.8)	583 (73.6)
Squamous	1223 (30.2)	209 (26.4)
ECOG PS, *n* (%)		
0–1	2866 (70.8)	792 (100.0)
≥2	1183 (29.2)	0
Race, *n* (%)		
White	3040 (75.1)	579 (73.1)
Black	445 (11.0)	17 (2.1)
Hispanic or Latino	122 (3.0)	0
Asian	84 (2.1)	174 (22.0)
Other	358 (8.8)	22 (2.8)
BMI, *n* (%)		
Underweight	348 (8.6)	40 (5.1)
Healthy	1780 (44.0)	364 (46.0)
Overweight	1176 (29.0)	258 (32.6)
Obese	745 (18.4)	130 (16.4)
PD‐L1 expression, *n* (%)		
Negative	1550 (38.3)	359 (45.3)
Positive	2499 (61.7)	433 (54.7)
*KRAS* mutations, *n* (%)		
Negative	2958 (73.1)	602 (76.0)
Positive	1091 (26.9)	190 (24.0)
*EGFR* mutations, *n* (%)		
Negative	3913 (96.6)	703 (88.8)
Positive	136 (3.4)	89 (11.2)

Abbreviations: BMI, body mass index; bpm, beats per minute; ECOG PS, Eastern Cooperative Oncology Group performance status; EGFR, epidermal growth factor receptor; IQR, interquartile range; PD‐L1, programmed death‐ligand 1.

**TABLE 2:** Laboratory characteristics of patients included in the prognostic model

Characteristic	Real‐world database (*n* = 4049)	OAK trial (*n* = 792)
White blood cells, *n* (%)		
High	824 (20.4)	124 (15.7)
Low	323 (8.0)	26 (3.3)
Normal	2902 (73.3)	642 (81.1)
Hemoglobin, *n* (%)		
High	10 (0.2)	0
Low	2812 (69.4)	792 (100)
Normal	1227 (30.3)	0
Protein total, *n* (%)		
High	86 (2.1)	38 (4.8)
Low	700 (17.3)	55 (6.9)
Normal	3263 (80.6)	699 (88.3)
Creatinine, *n* (%)		
High	550 (13.6)	101 (12.8)
Low	707 (17.5)	57 (7.2)
Normal	2792 (69.0)	634 (80.1)
Bilirubin, *n* (%)		
High	70 (1.7)	7 (0.9)
Low	361 (8.9)	28 (3.5)
Normal	3618 (89.4)	757 (95.6)
Aspartate aminotransferase, *n* (%)		
High	332 (8.2)	64 (8.1)
Low	197 (4.9)	15 (1.9)
Normal	3520 (83.4)	713 (90.0)
Alanine transaminase, *n* (%)		
High	230 (5.7)	40 (5.1)
Low	279 (6.9)	21 (2.7)
Normal	3540 (87.4)	731 (92.3)
Alkaline phosphatase, *n* (%)		
High	788 (19.5)	153 (19.3)
Low	39 (1.0)	11 (1.4)
Normal	3222 (79.6)	628 (79.3)
Lactate dehydrogenase, *n* (%)		
High	1599 (39.5)	336 (42.4)
Low	151 (3.7)	16 (2.0)
Normal	2299 (56.8)	440 (55.6)
Calcium, *n* (%)		
High	153 (3.8)	791 (99.9)
Low	518 (12.8)	1 (0.1)
Normal	3378 (83.4)	0
Chloride, *n* (%)		
High	97 (2.4)	22 (2.8)
Low	823 (20.3)	117 (14.8)
Normal	3129 (77.3)	653 (82.4)
Albumin, *n* (%)		
Abnormal (low)	1352 (33.4)	138 (17.4)
Normal/high	2697 (66.6)	654 (82.6)
Urea, *n* (%)		
High	627 (15.5)	789 (99.6)
Low	208 (5.1)	0
Normal	3214 (79.4)	3 (0.4)
Absolute eosinophil count, *n* (%)		
High	160 (4.0)	45 (5.7)
Low	67 (1.7)	45 (5.7)
Normal	3822 (94.4)	702 (88.6)
Monocyte, *n* (%)		
High	951 (23.5)	163 (20.6)
Low	89 (2.2)	7 (0.9)
Normal	3009 (74.3)	622 (78.5)
Lymphocyte, *n* (%)		
High	41 (1.0)	3 (0.4)
Low	1263 (31.2)	218 (27.5)
Normal	2745 (67.8)	571 (72.1)
Neutrophil, *n* (%)		
High	996 (24.6)	125 (15.8)
Low	84 (2.1)	10 (1.3)
Normal	2969 (73.3)	657 (83.0)
Platelet, *n* (%)		
High	406 (10.0)	83 (10.5)
Low	459 (11.3)	29 (3.7)
Normal	3184 (78.6)	680 (85.9)
Thrombocytosis, *n* (%)		
No	3643 (90.0)	709 (89.5)
Yes	406 (10.0)	83 (10.5)

### 3.2 Prognostic model

**FIGURE 2:**
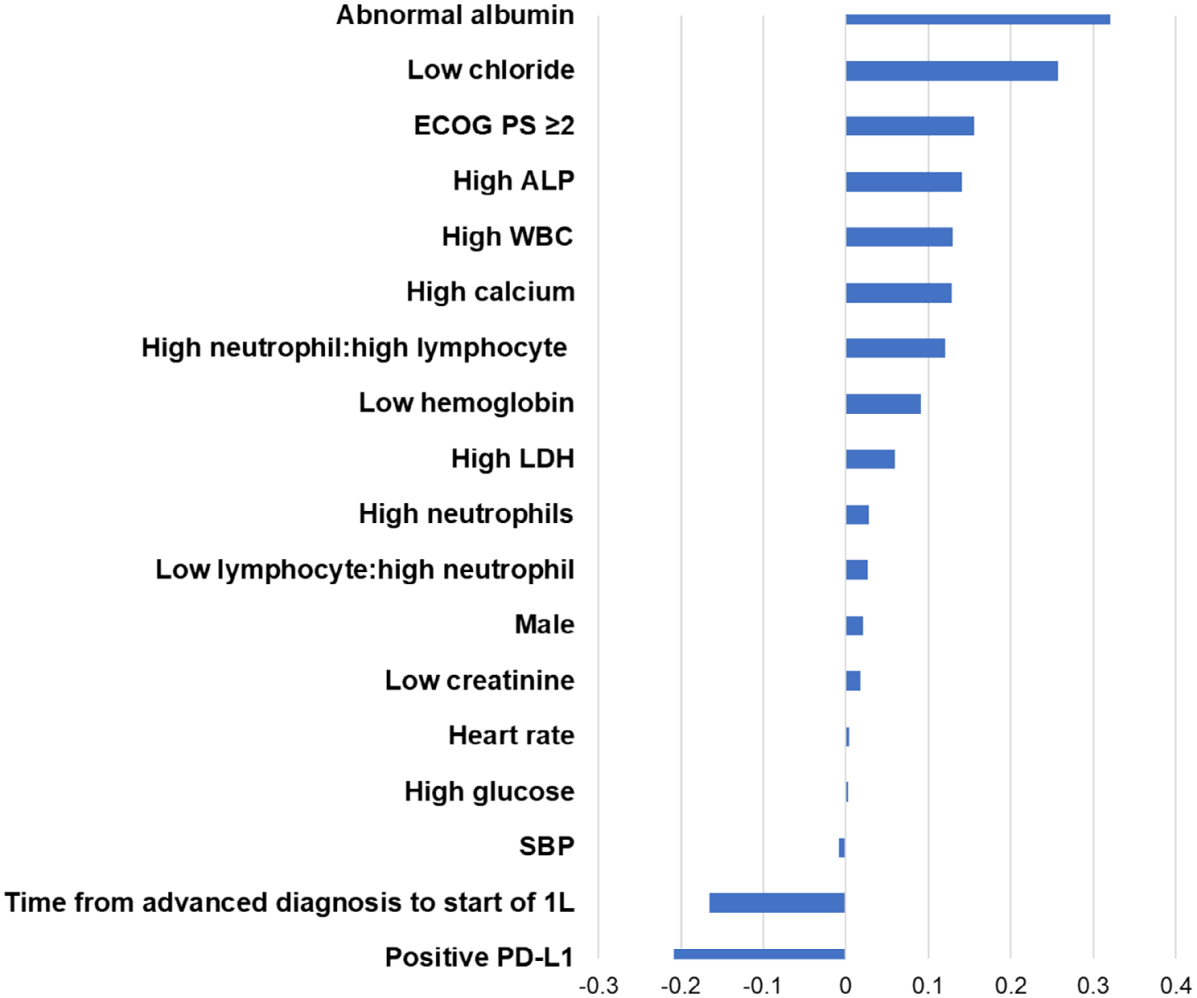
Summary of selected model coefficients. 1L, first line; ALP, alkaline phosphatase; ECOG PS, Eastern Cooperative Oncology Group performance status; LDH, lactate dehydrogenase; PD‐L1, programmed death‐ligand 1; SBP, systolic blood pressure; WBC, white blood cell

The final model selected 18 variables (Figure 2). The top risk‐increasing prognostic factors were abnormally low albumin and chloride levels, ECOG PS ≥2, and abnormally high levels of ALP and white blood cells. The top risk‐decreasing prognostic factors were PD‐L1 positivity, longer time from advanced diagnosis to start of 1L, and higher systolic blood pressure.

### 3.3 Model performance

**FIGURE 3:**
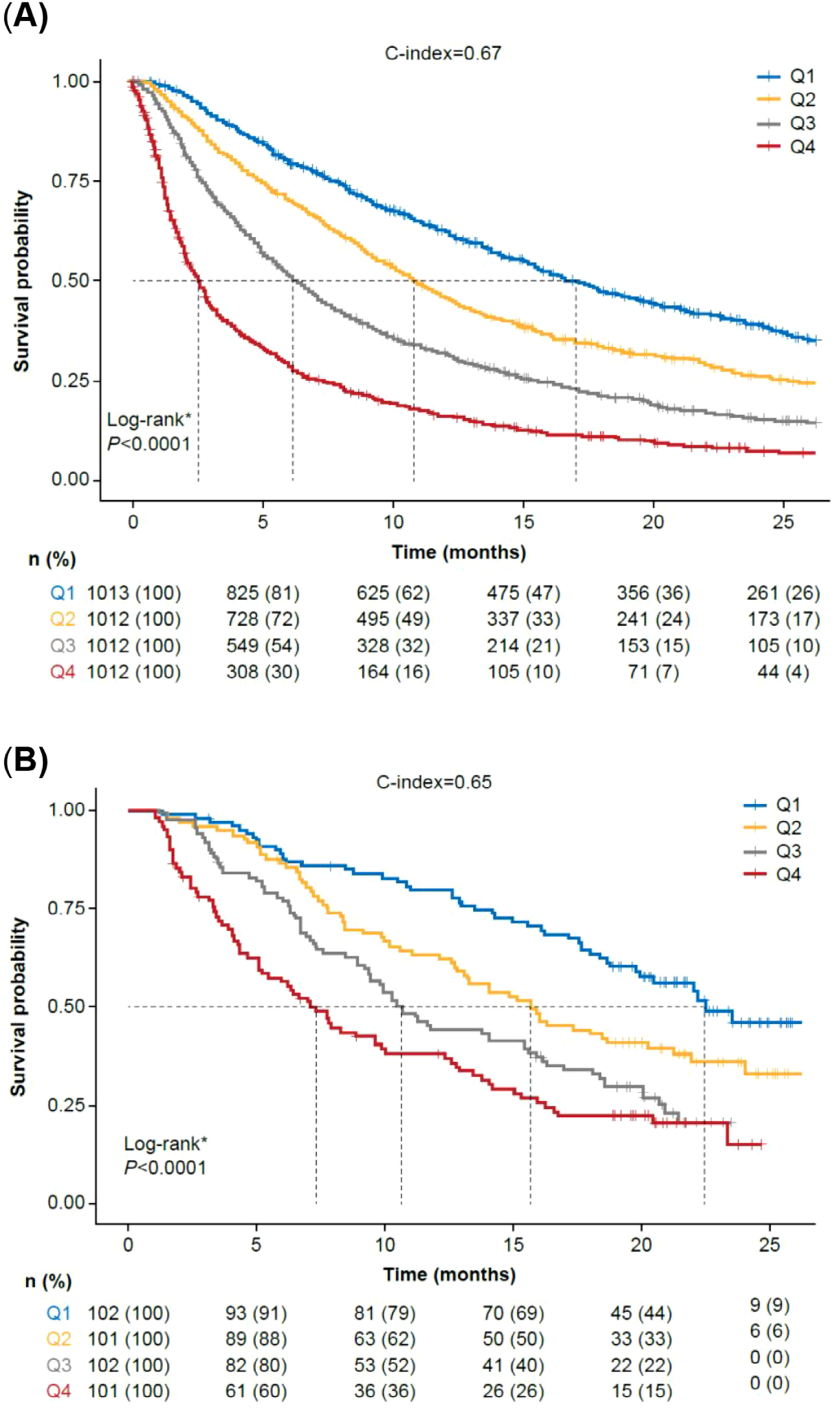
Association of prognostic index quartiles and overall survival in patients from (A) the real‐world database (derivation data) and (B) the OAK trial (validation data). Q1 represents the highest PI and Q4 represents the lowest PI. *The log‐rank test evaluated the null hypothesis of no difference in survival between the different PI groups. PI, prognostic index

When looking at the association of OS with the PI quartiles in both cohorts, OS was clearly differentiated among the PI quartiles (Figure 3). The c‐index calculated for the RWD cohort OS curves was 0.67, and the c‐index for the OAK trial validation cohort was 0.65. When using only ECOG PS to calculate the c‐index for the RWD, it was 0.56. Performance was similar across the CPI molecules (atezolizumab, nivolumab, and pembrolizumab; Figure S1).

## 4 DISCUSSION

To our knowledge, this is the first study developing a prognostic model in patients with aNSCLC receiving anti‐PD‐1/PD‐L1 CPIs as 2L monotherapy. This study evaluated factors that may influence patient survival and found several baseline demographic and clinical characteristics of prognostic value. A Cox proportional hazards model with lasso regularization was used to estimate the prognostic variables. This method limits overfitting due to either collinearity of the covariates or high dimensionality. The model was then validated using data from the OAK clinical trial.

This approach of evaluating multiple prognostic factors was improved compared with only using single prognostic factors, such as ECOG PS. The c‐index for ECOG PS was 0.56, whereas the c‐index of the prognostic model that incorporated multiple variables was 0.67. Compared with ROPRO, this model specifically focused on patients with aNSCLC receiving anti‐PD‐1/PD‐L1 CPIs as 2L monotherapy vs a more general, pooled cohort of 17 different tumor types who had received 1L treatment.^9^ This model incorporated 18 variables of which most were not defined by units of measurement (Figure 2) versus 27 variables defined by specific units in ROPRO.^9^ For laboratory values, levels were defined qualitatively (e.g., high vs. low) in this prognostic model compared with deriving a score based on the weighted sum of the patients' differences from the respective reference mean for each variable as performed in ROPRO.^9^ Simplifying how variables were defined (units or not) and the total number required could make this prognostic model easier to implement in clinical practice.

Risk‐decreasing factors included PD‐L1 positivity, longer time to advanced diagnosis, and higher systolic blood pressure. Being PD‐L1 positive was shown to decrease the risk, and patients who are PD‐L1 positive are known to respond better to PD‐1/PD‐L1 therapies.^19^ Recent research suggested that this could be a key factor for reducing cancer progression with immune checkpoint inhibition.^20^ Longer time from advanced diagnosis to start of 1L treatment was recognized as a risk‐decreasing factor. This finding should be interpreted with caution, as it is possible that the model assigned lower risk to patients having proved that they can survive for some time. This should not be interpreted as an argument for starting treatment later, because this is not a causal relationship. A higher systolic blood pressure was also observed as a risk‐decreasing factor. Previous studies have evaluated the use of hypertension treatments, such as angiotensin‐converting enzyme inhibitors, and the risk of cancer.^21^, ^22^ It could be hypothesized that systolic blood pressure and/or the use of hypertension treatments could be associated with cancer survival; although this has not been evaluated in the literature. When evaluating prognostic factors that were risk increasing, ECOG PS ≥2 was associated with a worse PI. This finding was not surprising, as ECOG PS is a well‐known prognostic factor of cancer and indicative of a patient's frailty, comorbidity, disease progression, and effects on daily life.^23^ Laboratory characteristics that were strong risk‐increasing prognostic factors included high ALP and white blood cell counts, and abnormally low albumin and low chloride levels. Both ROPRO and this model identified high ALP as a risk‐increasing prognostic factor, and ROPRO also noted that higher levels of albumin and chloride were protective, which aligns with this model's findings.^9^ Other index models have also recognized high white blood cell counts and ALP levels as well as low albumin levels as prognostic risk factors.^23^, ^24^, ^25^, ^26^ A retrospective analysis with a multivariate logistic regression analysis indicated that ALP was an independent risk factor for bone metastases in patients with bladder cancer.^27^ A retrospective study in Turkish patients with aNSCLC (stage IIIB) found that low pretreatment serum albumin level was an independent poor prognostic factor in patients with aNSCLC, which was associated with a reduced response to 1L therapy and decreased survival rates.^28^ A systematic review also reported that low pretreatment serum albumin was associated with poor survival and could be used to define baseline patient risk.^24^ Only limited studies have explained the low chloride findings in aNSCLC. In colorectal cancer, a retrospective, single‐center study found that hypochloremia was associated with shorter OS and disease‐free survival in patients with early‐ to later‐stage disease who had resection.^29^ Further research is warranted to understand the role of chloride levels in aNSCLC outcomes.

It is important to highlight that extracting clinically relevant data using electronic health records is a complex process and is a limitation of the retrospective nature of the present study. This database lacked complete information on other possible prognostic factors such as C‐reactive protein, steroid use, tumor‐infiltrating lymphocytes, pro‐inflammatory cytokine levels, and T‐cell (CD3+), cytotoxic T‐cell, and memory T‐cell counts. For laboratory‐based data, values were categorized based on the reference range supplied by the laboratory in the real‐world setting. These ranges can vary across laboratories, and accordingly, “cutoff” minimum and maximum values may differ across methods/patients. While laboratory‐to‐laboratory variability exists, this heterogeneity can allow more flexibility when implementing the prognostic model in populations in which different instruments, techniques, or reference ranges have been used across multiple clinical sites.

In summary, these analyses used a large cohort of patients with aNSCLC from a real‐world setting to create a prognostic model, which was validated using clinical trial data. By using baseline demographic, clinical, and laboratory factors, this prognostic model could discriminate the risk of death among patients with aNSCLC receiving anti‐PD1/PDL1 CPIs in 2L therapy. Further research into prognostic models to aid treatment selection is a critical element of ensuring optimal support for decision‐making in real practice.

## CONFLICT OF INTEREST

Cristina Julian, Pascal Chanu, Chris Harbron, Anda Gershon, Dominik Heinzmann, Wei Zou, and Valerie Quarmby are employees and shareholders of Genentech/Roche. Cristina Julian, Robson J. M. Machado, Sandhya Girish, Pascal Chanu, Dominik Heinzmann, Chris Harbron, Valerie Quarmby, Qing Zhang, and Yachi Chen have a planned/issued/pending patent with Roche. Chris Harbron also owns stock in AstraZeneca. Shannon M. Pfeiffer received payment from Genentech for a 10‐month internship. Yachi Chen, Robson J. M. Machado, and Sandhya Girish were employees of Genentech/Roche at the time of the study; Yachi Chen is a shareholder of Genentech/Roche. Qing Zhang is an employee of Genentech/Roche and owns stock in AbbVie, AC Immune, Bristol Myers Squibb, Regeneron, and Roche.

## AUTHOR CONTRIBUTIONS

All authors had full access to the data in the study and take responsibility for the integrity of the data and the accuracy of the data analysis. *Conceptualization, Formal analysis, Investigation, Methodology, Project administration, Supervision, Validation, Visualization, Writing‐original draft, Writing‐review & editing*, C.J.; *Conceptualization, Data curation, Formal analysis, Investigation, Methodology, Project administration, Software, Supervision, Validation, Visualization, Writing‐original draft, Writing‐review & editing*, R.J.M.M.; *Conceptualization, Investigation, Project administration, Supervision, Visualization, Writing‐original draft, Writing‐review & editing*, S.G.; *Conceptualization, Investigation, Methodology, Project administration, Supervision, Visualization, Writing‐original draft, Writing‐review & editing*, P.C.; *Conceptualization, Formal analysis, Funding acquisition, Investigation, Methodology, Validation, Visualization, Writing‐original draft, Writing‐review & editing*, D.H.; *Formal analysis, Methodology, Writing‐original draft, Writing‐review & editing*, C.H.; *Conceptualization, Investigation, Methodology, Project administration, Supervision, Validation, Visualization, Writing‐original draft, Writing‐review & editing*, A.G.; *Formal analysis, Methodology, Software, Writing‐original draft, Writing‐review & editing*, S.M.P.; *Conceptualization, Writing‐original draft, Writing‐review & editing*, W.Z.; *Writing‐original draft, Writing‐review & editing*, V.Q.; *Conceptualization, Data curation, Funding acquisition, Investigation, Methodology, Project administration, Resources, Supervision, Visualization, Writing‐original draft, Writing‐review & editing*, Q.Z.; *Conceptualization, Methodology, Supervision, Writing‐original draft, Writing‐review & editing*, Y.C.

## ETHICS STATEMENT

Institutional review board approval of the study protocol was obtained prior to study conduct and included a waiver of informed consent. The data are deidentified and subject to obligations to prevent re‐identification and protect patient confidentiality.

## Supporting information


**Supplemental Figure 1.** Association of PI quartiles and overall survival in patients from the real‐world database for (A) atezolizumab, (B) nivolumab, and (C) pembrolizumab. Q1 represents the highest PI and Q4 represents the lowest PI. * The log‐rank test evaluated the null hypothesis of no difference in survival between the different PI groups. PI, prognostic index.

## Data Availability

The data that support the findings of this study have been originated by Flatiron Health, Inc. These deidentified data may be made available upon request and are subject to a license agreement with Flatiron Health; interested researchers should contact <dataaccess@flatiron.com> to determine licensing terms. Qualified researchers may request access to individual patient‐level data through the clinical study data request platform (https://vivli.org/). Further details on Roche's criteria for eligible studies are available here (https://vivli.org/members/ourmembers/). For further details on Roche's Global Policy on the Sharing of Clinical Information and how to request access to related clinical study documents, see (https://www.roche.com/research_and_development/who_we_are_how_we_work/clinical_trials/our_commitment_to_data_sharing.htm).
