# Supplementary material for: Real‐world data prognostic model of overall survival in patients with advanced NSCLC receiving anti‐PD‐1/PD‐L1 immune checkpoint inhibitors as second‐line monotherapy
Source: Cancer Rep (Hoboken). 2022 Jan 24;5(10):e1578. doi: 10.1002/cnr2.1578 (PMC9575492; doi:10.1002/cnr2.1578)
Supplement: Supplementary file 1 — Supplemental Figure 1. Association of PI quartiles and overall survival in patients from the real‐world database for (A) atezolizumab, (B) nivolumab, and (C) pembrolizumab. Q1 represents the highest PI and Q4 represents the lowest PI. * The log‐rank test evaluated the null hypothesis of no difference in survival between the different PI groups. PI, prognostic index. [file CNR2-5-e1578-s001.docx]

**SUPPLEMENTAL FIGURES**

**Supplemental Figure 1.** Association of PI quartiles and overall survival in patients from the real-world database for (A) atezolizumab, (B) nivolumab, and (C) pembrolizumab. Q1 represents the highest PI and Q4 represents the lowest PI. * The log-rank test evaluated the null hypothesis of no difference in survival between the different PI groups. PI, prognostic index.

**A.**

**B.**

**C.**
